# Pterosaurs ate soft-bodied cephalopods (Coleoidea)

**DOI:** 10.1038/s41598-020-57731-2

**Published:** 2020-01-27

**Authors:** R. Hoffmann, J. Bestwick, G. Berndt, R. Berndt, D. Fuchs, C. Klug

**Affiliations:** 1 Institut für Geologie, Mineralogie und Geophysik, Ruhr-Universität Bochum, Universitätsstrasse 150, 44801 Bochum, Germany; 2 Centre for Palaeobiology Research, School of Geography, Geology & the Environment, University of Leicester, Leicester, LE1 7RH UK; 3 Unaffiliated, Berlin, Germany; 4 Unaffiliated, Dortmund, Germany; 5 SNSB-Bayerische Staatssammlung für Paläontologie und Geologie, Richard-Wagner-Strasse 33, 80333 Munich, Germany; 6 Paläontologisches Institut und Museum, Universität Zürich, Karl Schmid-Strasse 4, CH-8006 Zürich, Switzerland

**Keywords:** Palaeontology, Palaeoecology

## Abstract

Direct evidence of successful or failed predation is rare in the fossil record but essential for reconstructing extinct food webs. Here, we report the first evidence of a failed predation attempt by a pterosaur on a soft-bodied coleoid cephalopod. A perfectly preserved, fully grown soft-tissue specimen of the octobrachian coleoid *Plesioteuthis subovata* is associated with a tooth of the pterosaur *Rhamphorhynchus muensteri* from the Late Jurassic Solnhofen Archipelago. Examination under ultraviolet light reveals the pterosaur tooth is embedded in the now phosphatised cephalopod soft tissue, which makes a chance association highly improbable. According to its morphology, the tooth likely originates from the anterior to middle region of the upper or lower jaw of a large, osteologically mature individual. We propose the tooth became associated with the coleoid when the pterosaur attacked *Plesioteuthis* at or near the water surface. Thus, *Rhamphorhynchus* apparently fed on aquatic animals by grabbing prey whilst flying directly above, or floating upon (less likely), the water surface. It remains unclear whether the *Plesioteuthis* died from the pterosaur attack or survived for some time with the broken tooth lodged in its mantle. Sinking into oxygen depleted waters explains the exceptional soft tissue preservation.

## Introduction

Constraining the diets of extinct taxa is vital for understanding predator-prey relationships, reconstructing extinct food webs and for understanding the evolution of multi-trophic interactions^1–4^. Fossilised gut and throat contents, known as content fossils^4^, are perhaps the most renowned line of direct evidence for extinct predator-prey interactions. These fossils have greatly increased the known dietary ranges of many extinct clades, including carnivory in Mesozoic mammals^5^ and piscivory in theropod dinosaurs^6^. However, well-preserved content fossils are extremely rare and there is an inbuilt bias towards preservation of ‘harder’ items (e.g. scales and shells) and towards items consumed immediately prior to death^4,7^.

Other direct lines of evidence used to infer diets include coprolites^8^, regurgitalites^9^, tooth marks from supposed feeding events^10,11^, healed bite traces from failed predation attempts^12^ (which sometimes contain embedded teeth) and the preservation of predators and prey together as a result of fatal encounters^13^. These types of evidence are slightly more abundant than content fossils and are also useful for gaining unique insights into the foraging and feeding behaviours and the habitat preferences of both predators and prey^1^. For example, the presence of bone regrowth in the damaged caudal neural spines in the hadrosaurid dinosaur *Edmontosaurus* has been interpreted as evidence of active predation by the large theropod dinosaur *Tyrannosaurus rex* and that *T. rex*, consequently, was not an obligate scavenger^12^. Inferring the taxonomic identities of predators and prey from these types of evidence, however, can be difficult^1,14^.

Well-preserved evidence of predator-prey relationships is primarily known from Konservat-Lagerstätten as rapid specimen burial prevents carcass damage from scavengers and/or anoxic conditions facilitates soft-tissue preservation^15,16^. For example, the Late Jurassic Solnhofen Archipelago, Germany, contains lagoons with low oxygen concentrations and high salinities^17^, which enabled the phosphatization of soft tissues^18–21^. From these Late Jurassic plattenkalks, numerous coleoids with preserved phosphatized and carbonized soft tissues such as the arms, fins, gills, digestive tract, ink sac and duct, cephalic cartilage with eye capsules plus statocysts, stomach content, mantle and spermatophores have been reported^22–32^. Lagerstätten such as Solnhofen therefore yield primary data, which allow inferences on the diets and ecological roles of soft-bodied organisms and ultimately on ancient ecosystems and foodwebs. This is usually impossible in fossils from other deposits.

One such clade are Mesozoic coleoid cephalopods, which are related to modern squid and sepiids. These fossils are known almost entirely from Konservat-Lagerstätten (conservation deposits). Mesozoic coleoids, such as *Plesioteuthis*, have repeatedly been inferred to have been active predators based on several lines of independent evidence. These lines of reasoning include (i) fossilised stomach contents containing ammonoid and fish remains^33–35^, (ii) a slender body, squid-like mantle and four fins that reportedly enabled fast swimming and provided a good manoeuvrability^25,36–39^, and (iii) other predator-prey associations^33,40,41^. However, direct evidence of extinct coleoids as prey has, until now, been rare^35,42–44^.

**Figure 1:**
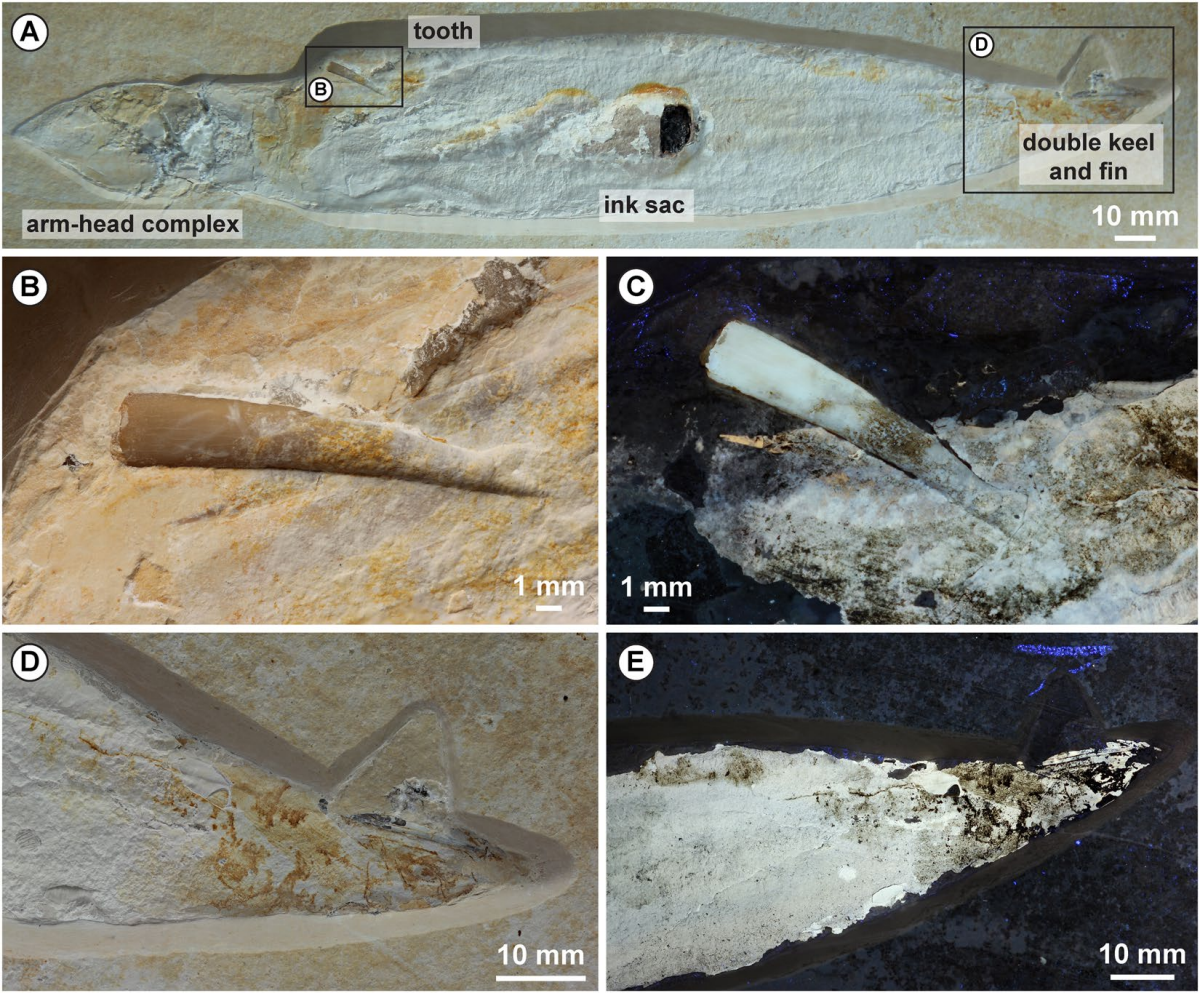
*Plesioteuthis subovata* from the Late Jurassic Solnhofen Archipelago. An adult specimen, 28 cm long, preserved with ink sac and duct, arm-head complex, well-preserved mantle musculatures (transverse striation) and a pterosaur tooth. (**B**) Close-up of the 19 mm long, slightly curved *Rhamphorhynchus muensteri* tooth crown under normal light. (**C**) Ultraviolet (UV) light reveals that the tooth apex is partially covered with now phosphatized mantle tissue. (**D**) Posterior part of the specimen with faint imprints probably representing a terminal fin, but UV light provides no evidence of fin musculature (**E**). The posterior mantle margin is incomplete and a straight structure with a double keel typical for *P. subovata* provides evidence of the gladius. All photographs by J. Härer, used with permission.

Here, we document the first case of an exceptionally preserved coleoid cephalopod, *Plesioteuthis*, from the Solnhofen Archipelago that is preserved with an associated pterosaur tooth (Fig. 1). We examine the ecological relationship between soft-bodied coleoid cephalopods as prey and pterosaurs, such as *Rhamphorhynchus*, as a non-marine predator of marine organisms. We describe the material in detail and discuss their taphonomy and the ecological aspects of both animals. This includes possible feeding behaviours of *Rhamphorhynchus* and the likely habitat of *Plesioteuthis*.

## Description

One specimen of the octobrachian cephalopod *Plesioteuthis subovata* is available preserving a pterosaur tooth of *Rhamphorhynchus muensteri* stuck in its phosphatized mantle musculature. The specimen is kept in the Paläontologisches Institut und Museum, Universität Zürich, Switzerland (PIMUZ 37358). It was collected by GB and RB in 2012 from the Solnhofen Archipelago (Blumenberg near Eichstätt) in southeastern-Germany from the lower Tithonian (Late Jurassic) Altmühltal Formation (*Hybonoticeras hybonotum* zone). The specimen was prepared by Udo Resch (Eichstätt) and GB.

### *Plesioteuthissubovata*

The specimen has a total body length of 285 mm, 188 mm of which comprises the mantle (=gladius; Fig. 1). The strongly flattened specimen is seen in ventral view, i.e., the dorsally located gladius is covered by the muscular mantle, whose circular muscles are visible as fine transversal *striae* perpendicular to the longitudinal body axis. In several Konservat-Lagerstätten, such as the Posidonia Shale, Oxford Clay and Solnhofen Archipelago, the mantle musculature is commonly phosphatized and preserved as whitish to brownish apatite^26^.

The arm-head complex is clearly demarcated from the mantle. As usual in the Solnhofen plattenkalks, a sparitic calcitic concretion is formed near the buccal mass, destroying fine anatomical detail. Musculature remains of the two arm stubs are preserved anterior to this septaria. Anterior-most imprints suggest a total arm length of about 37 mm, indicating an arm length to mantle length ratio of 0.20. Neither suckers nor cirri are visible.

The well-preserved mantle musculature shows the characteristic transverse striation and the elongate, formerly cigar-shaped mantle. The ink sac and duct are visible as bulges in the mantle. Fossilized black ink can be seen in a small hole where the phosphatized mantle broke off. Musculature of the ventral funnel covering the ink duct is also preserved. The faint imprints at the right posterior end of the specimen probably represent a terminal fin, but ultraviolet (UV) light provides no evidence of fin musculature (proportions and position support this interpretation). The posterior mantle margin is incomplete and a straight structure with a double keel provides evidence of the gladius.

**Figure 2:**
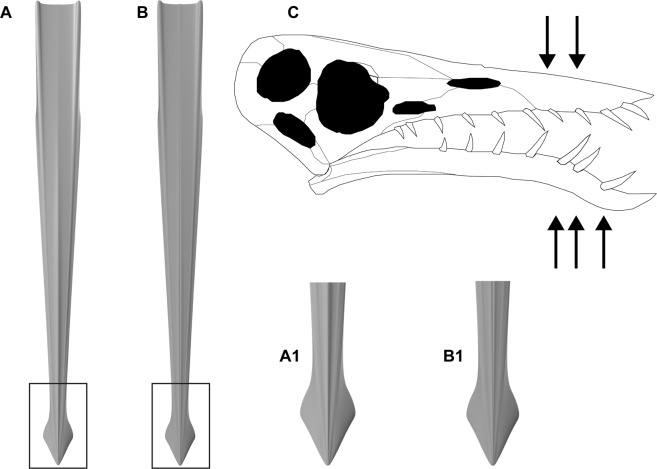
Cephalopod gladius and pterosaur skull reconstructions. (**A**) *Plesioteuthis prisca* with an unipartite median keel. (**B**) *Plesioteuthis subovata* with a bipartite median keel. (**C**) *Rhamphorhynchus* skull with upper and lower jaw dentition (black arrows indicate potential position of the broken off tooth that became stuck in the *Plesioteuthis* mantle tissue). Skull drawing after Bennett^73^ reproduced with permission.

The cigar-shaped mantle and the short arms are typical for *Plesioteuthis prisca*. However, in *P. prisca* the median keel is unipartite in contrast to the present specimen (Fig. 2). Such a bipartite keel is typical for *P. subovata*^32,36,39^. Unlike *P. prisca*, *P. subovata* is one of the rarest coleoids of the Solnhofen Archipelago, thus underlining the uniqueness of this fossil. In *Plesioteuthis*, almost all non-mineralized tissues and organs have been preserved in at least one specimen^22,32,33,36,45–48^. Several anatomical details (e.g., eight arms, uniserial suckers without sucker rings, shape of the jaws, presence of four fins) suggest that Mesozoic gladius-bearing coleoids unambiguously belong to the octobrachian lineage, summarised by Donovan & Fuchs^26^.

### *Rhamphorhynchus* tooth

The tooth perforated the left flank of the anterior mantle margin (Fig. 1A). The tip of the tooth is stuck in the mantle margin (which is somewhat stiffer than the rest of the mantle) but has probably not reached the internal organs. There is no evidence of additional injuries. The embedded tooth is approximately 19 mm long (only the crown is preserved) and 3 mm wide at its base (height/width ratio; 6.33). The tooth is slender, laterally compressed, and gently recurved, especially close to the pointed tip. Much of the enamel near the tip of the tooth is covered by phosphatized mantle (Fig. 1C). However, the brown patches around the centre of the tooth, revealed by UV light, may represent enamel. The tooth’s dentine is light brown in colour in the basal half of the tooth under white light and is white under UV light. No serrations, carinae or longitudinal ridges are visible, but thin longitudinal cracks in the dentine are visible.

## Discussion

A large number of taxa from the Solnhofen Archipelago, including ‘fishes’ and marine reptiles, supposedly fed on aquatic organisms such as cephalopods^4,9,49–52^. The conical, slender and recurved shape of the tooth excludes several groups of fish and reptiles as possible culprits. Holostean and pycnodontiform fishes (both Actinopterygii), for example, both possess rounded, hemispherical teeth^51,53,54^. Chondrichthyans, i.e. sharks, rays and chimaeras, possess either serrated, multi-cusped teeth or flattened tooth-plates^54,55^. The tooth morphology also excludes aquatic rhynchocephalian reptiles such as *Pleurosaurus*, which possess triangular, anteroposteriorly elongate teeth^56^.

Other marine reptiles such as pliosaurs, ichthyosaurs and metriorhynchid as well as teleosaurid crocodyliformes have conical teeth^49,57^. However, the teeth of most of these reptiles display longitudinal ridges^49,57,58^, which are not developed in our specimen. The relatively high preservation quality of the dentine indicates that the absence of ridges in our specimen is primary rather than an artefact. The teeth of some metriorhynchid and teleosaurid crocodyliformes lack longitudinal ridges^59^. These teeth still differ because they generally exhibit height/ width ratios of around 2–3^49^, which are much stouter than our specimen, although they are slender compared to other crocodyliformes. This, in combination with the great agreement of its shape with that of pterosaur teeth, support a pterosaur as the likely culprit. Of the known Solnhofen pterosaurs, the tooth shape is most similar to that of *Rhamphorhynchus muensteri*^60,61^. *Rhamphorhynchus* teeth usually have well-developed carinae along the anterior and posterior margins^62^, but our specimen is embedded laterally and thus, such carinae would be obscured by the surrounding mantle tissue. The possible enamel-dentine boundary around the tooth centre contrasts slightly with *Rhamphorhynchus* teeth where the boundary has been described around one-third of the way down the tooth from the tip^60,62^. However, the upper third of the tooth is covered by the cephalopod mantle and thus prevents conclusive identification of the enamel-dentine boundary (this would also be difficult to see in CT since both the tooth and the surrounding cephalopod mantle is composed of apatite). Based on its size, height-to-width ratio and curvature, the tooth was most likely from the anterior to middle region of the upper or lower jaw (Fig. 2C). This also makes sense from ecological and mechanical points of view as the anterior teeth are the most likely to come into regular contact with food items and are subjected to higher bending movements while biting struggling prey^62^. These teeth therefore have the highest likelihood of breakage during feeding^62^.

Furthermore, the nature of the *Rhamphorhynchus* fossil record allow us to estimate the likely ontogenetic stage of the perpetrator. The teeth of hatchling and immature juveniles are very small and straight (e.g., Wellnhofer^63^; pp. 81–82), while the teeth of the largest adults have a lower height-to-width ratio and slightly blunt tips (e.g., Bonde & Leal^61^; Fig. 5a). Accordingly, the tooth stuck in the coleoid likely belonged to a large, osteologically mature individual (over 1 metre wingspan).

Interpreting ecological relationships between extinct taxa based on the spatial association of specimens can be flawed if taphonomic biases, such as post-mortem specimen transport, are not taken into consideration^4,64^. However, abiotic explanations for the association of *Plesioteuthis* and *Rhamphorhynchus* can be ruled out for several reasons:

1. As reflected in the perfectly horizontally bedded sediments, the lagoons of the Solnhofen Archipelago are generally regarded as a low energy depositional environment barring storm events^52^. Specimens are therefore unlikely to have been transported far along the sea floor.
2. The absence of a root suggests that the tooth broke off at the crown-root boundary.
3. The tooth penetrated the *Plesioteuthis* mantle, which is highly unlikely to have occurred via transport in such a low energy depositional environment.

**Figure 3:**
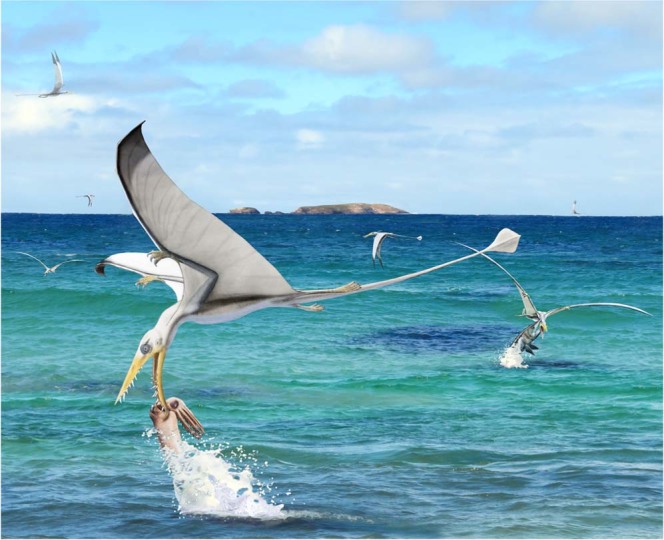
Reconstruction of the hunting behaviour of *Rhamphorhynchus muensteri*, flying close to the water surface to grab soft-bodied cephalopods such as *Plesioteuthis subovata* that lived in the uppermost part of the water column. Artwork and background photograph by CK and Beat Scheffold using a model produced by the latter (Zürich).

We therefore conclude that the *Rhamphorhynchus* tooth became associated with the *Plesioteuthis* through the pterosaur biting the cephalopod. At least one *Rhamphorhynchus* tooth broke during this interaction, which resulted in the *Plesioteuthis* escaping with a tooth remaining stuck in its mantle and an unsuccessful predation attempt. (Figure 3).

Direct evidence of pterosaurs as predators is currently restricted to fossil gut and throat contents. Fish, crustaceans and tetrapods have been identified from stomach contents from several pterosaur taxa^4,65^ (and references therein). Such stomach contents are most often preserved with *Rhamphorhynchus* and confidently identified remains are currently limited to fish^65^. A coprolite assigned to *Rhamphorhynchus* was interpreted to have contained coleoid arm hooklets^14^, although this interpretation has since been challenged^4^. Speculative cases of attempted pterosaur feeding involve odonatan insects from the Solnhofen Archipelago with damaged wings^65^ (and references therein). Our specimen therefore provides the first direct evidence of a pterosaur-prey interaction that did not result in prey consumption. It should be noted that until additional similar specimens are found, it remains impossible to rule out that the pterosaur misinterpreted the cephalopod as a different food item. Nevertheless, the specimen is important for understanding ecological interactions between Solnhofen taxa, and it is conceivable that pterosaurs were possibly at least somewhat opportunistic in their choice of prey. We also think that this specimen more likely reflects predatory behaviour from *Rhamphorhynchus* rather than scavenging behaviour. Coleoid cephalopods have been experimentally shown to decay rapidly after death as the dermis ruptures within the first 24–48 hours after death, which further facilitates the decay of the internal tissues^21,66^. The well-preserved nature of the musculature and head-arm complex in our specimen indicates that it was buried soon after death rather than floating at the water surface for a prolonged period of time where it would have been exposed to scavengers. Moreover, it is unlikely that pterosaur dived down to the anoxic bottom water layers to scavenge on carcasses. However, we are currently unable to say whether the *Plesioteuthis* died as a result of the pterosaur attack or survived for some time with the broken tooth stuck in its mantle.

There is a general lack of agreement concerning pterosaur feeding behaviour^4^. In the case of our specimen, some conclusions on behaviours can be drawn with some reservation. Accepting that the pterosaur attacked the coleoid near the water surface, this implies that these cephalopods lived primarily in the upper part of the water column of the Solnhofen Archipelago^52,67^. In addition, several *Rhamphorhynchus* fossils have been described where a specimen is entangled within the jaws of the predatory fish, *Aspidorhynchus*^13^. This encounter was stated to have happened near or at the water surface in order for an aquatic animal to successfully catch an aerial animal with its jaws^13^, indirectly corroborating our suggestion that the pterosaur-cephalopod interaction occurred near the water surface (Fig. 3). Skim-feeding has been suggested for *Rhamphorhynchus*^63,68^, but has since experimentally been shown to have been too energy expensive^69–71^. *Rhamphorhynchus* was therefore more likely to have fed on aquatic prey by grabbing prey whilst on the wing just above the water surface, or while floating on the water surface^65,72^.

## Conclusion

We describe an adult specimen of the extremely rare octobrachian coleoid cephalopod *Plesioteuthis subovata* preserved with a tooth of the pterosaur *Rhamphorhynchus muensteri* in its mantle tissue. We present this association as the first direct evidence of a predator-prey interaction between pterosaurs and cephalopods. This interaction took place at or near the water surface. A scavenging feeding mode for *Rhamphorhynchus* is doubtful because the pterosaur is unlikely to have dived to the highly dangerous anoxic sediment floor to access carrion. It is also unlikely that tooth breakage would occur while consuming the soft decaying mantle of a coleoid carcass. Most likely, the tooth broke off in the *Plesioteuthis* mantle when the pterosaur attacked and the cephalopod tried to escape. High mechanical stress was exerted to the base of the teeth that were in direct contact with the cephalopod. This fractured at least one tooth, which remained stuck in the mantle. It is impossible to assess whether the *Plesioteuthis* died as a result of the pterosaur attack or survived with the broken tooth in its mantle. In addition to revealing cephalopods as a likely part of the *Rhamphorhynchus* diet, this fossil provides evidence that *Plesioteuthis* commonly lived in the upper part of the water column where it was accessible to pterosaurs.

## References

[1] O’Connor J, Zhou Z, Xu X (2011). Additional specimen of *Microraptor* provides unique evidence of dinosaurs preying on birds. Proceedings of the National Academy of Sciences of the United States of America.

[2] O’Connor J (2019). *Microraptor* with ingested lizard suggested non-specialized digestive function. Current Biology.

[3] Hone DWE, Faulkes CG (2014). A proposed framework for establishing and evaluating hypotheses about the behavior of extinct organisms. Journal of Zoology.

[4] Bestwick J, Unwin DM, Butler RJ, Henderson DM, Purnell MA (2018). Pterosaur dietary hypotheses: a review of ideas and approaches. Biological Reviews.

[5] Hu Y, Meng J, Wang Y, Li C (2005). Large Mesozoic mammals fed on young dinosaurs. Nature.

[6] Charig A, Milner J (1997). A. C. *Baryonyx walkeri*, a fish-eating dinosaur from the Wealden of Surrey. Bulletin of the Natural History Museum of London Geological Series.

[7] Davis M, Pineda-Munoz S (2016). The temporal scale of diet and dietary proxies. Ecology and Evolution.

[8] Chin K (2002). Analyses of coprolites produced by carnivorous vertebrates. Paleontological Society Papers.

[9] Hoffmann R., Stevens K., Keupp H., Simonsen S., Schweigert G. (2019). Regurgitalites – a window into the trophic ecology of fossil cephalopods. Journal of the Geological Society.

[10] Currie PJ, Jacobsen AR (1995). An azhdarchid pterosaur eaten by a velociraptorine theropod. Canadian Journal of Earth Sciences.

[11] Jacobsen AR (1998). Feeding behavior of carnivorous dinosaurs as determined by tooth marks on dinosaur bones. Historical Biology.

[12] Carpenter, K. *Evidence of predatory behaviour by carnivorous dinosaurs. Aspects of theropod palaeobiology, Gaia*. (eds. Pérez-Moreno, B. P., Holtz, T., Sanz, J. L. & Moratalla, J. J.) Museu Nacional de História Natural, Lisbon **15**, 135–144 (1998).

[13] Frey E, Tischlinger H (2012). The Late Jurassic pterosaur *Rhamphorhynchus*, a frequent victim of the ganoid fish *Aspidorhynchus*?. PLoS One.

[14] Hone DWE, Henderson DM, Therrien F, Habib MB (2015). A specimen of *Rhamphorhynchus* with soft tissue preservation, stomach contents and a putative coprolite. PeerJ.

[15] Seilacher A (1970). Begriff und Bedeutung der Fossil-Lagerstätten. Neues Jahrbuch für Geologie und Paläontologie.

[16] Xing L (2012). Abdominal contents from two large Early Cretaceous compsognathids (Dinosauria: Theropoda) demonstrate feeding on confuciusornithids and dromaeosaurids. PLoS ONE.

[17] Reisdorf A, Wuttke M (2012). Re-evaluating Moodie´s Opisthonic-Posture Hypothesis in Fossil Vertebrates Part I: Reptiles-the taphonomy of the bipedal dinosaurs *Compsognathus longipes* and *Juravenator starki* from the Solnhofen Archipelago (Jurassic, Germany). Palaeobiology and Palaeoenvironment.

[18] Allison PA (1988). Konservat-Lagerstätten: cause and classification. Paleobiology.

[19] Briggs DEG, Kear AJ, Martill DM, Wilby PR (1993). Phosphatization of soft-tissue in experiments and fossils. Journal of the Geological Society, London.

[20] Briggs DEG, Wilby PR (1996). The role of the calcium carbonate-calcium phosphate switch in the mineralization of soft-bodied fossils. Journal of the Geological Society, London.

[21] Kear AJ, Briggs DE, Donovan DT (1995). Decay and fossilization of non-mineralized tissue in coleoid cephalopods. Palaeontology.

[22] Fuchs D (2006). Fossil erhaltungsfähige Merkmalskomplexe der Coleoidea (Cephalopoda) und ihre phylogenetische Bedeutung. Berliner Paläobiologische Abhandlungen.

[23] Fuchs DD (2006). taxonomy and morphology of vampyropod Coleoids (Cephalopoda) from the Upper Cretaceous of Lebanon. Memorie della Società Italiana di Scienze Naturali et del Museo Civico di Storia Naturale di Milano.

[24] Fuchs D, Keupp H, Engeser T (2003). New records of soft parts of *Muensterella scutellaris* MUENSTER, 1842 (Coleoidea) from the Late Jurassic Plattenkalks of Eichstätt and their significance for octobrachian relationships. Berliner Paläobiologische Abhandlungen.

[25] Fuchs D, Iba Y, Tischlinger H, Keupp H, Klug C (2016). The locomotion system of fossil Coleoidea (Cephalopoda) and its phylogenetic significance. Lethaia.

[26] Donovan DT, Fuchs D, Part M (2016). Chapter 10: Fossilized soft tissues in Coleoidea. Treatise Online.

[27] Dietl G, Schweigert G (1999). Ein Nautilus mit *in-situ* liegendem, vollständigem Kieferapparat aus dem Nusplinger Plattenkalk (Oberjura, SW-Deutschland). Neues Jahrbuch für Geologie und Paläontologie.

[28] Schweigert G, Dietl G (1999). Zur Erhaltung und Einbettung von Ammoniten im Nusplinger Plattenkalk (Oberjura, Südwestdeutschland). Stuttgarter Beiträge zur Naturkunde, Serie B (Geologie und Paläontologie).

[29] Schweigert G, Dietl G (2001). Die Kieferelemente von *Physodoceras* (Ammonitina, Aspidoceratidae) im Nusplinger Plattenkalk (Oberjura, Schwäbische Alb). Berliner geowiss. Abh., Reihe E.

[30] Schweigert G, Dietl G (2008). Miscellanea aus dem Nusplinger Plattenkalk (Ober-Kimmeridgium, Schwäbische Alb). 9. Eine neue Aptychen-Formgattung. Jahresberichte und Mitteilungen des Oberrheinischen geologischen Vereins, Neue Folge.

[31] Klug C, Schweigert G, Dietl G, Fuchs D (2005). Coleoid beaks from the Nusplingen Lithographic Limestone (Late Kimmeridgian, SW Germany). Lethaia.

[32] Klug C, Schweigert G, Dietl G (2010). A new *Plesioteuthis* with beak from the Kimmeridgian of Nusplingen (Germany). Ferrantia.

[33] Keupp H, Engeser T, Fuchs D, Haeckel W (2010). Ein *Trachyteuthis hastiformis* (Cephalopoda, Coleoidea) mit Spermatophoren aus dem Ober-Kimmeridgium von Painten (Ostbayern). Archaeopteryx.

[34] Fuchs D, Larson NL (2011). Diversity, morphology, and phylogeny of coleoid cephalopods from the Upper Cretaceous plattenkalks of Lebanon - Part I: Prototeuthidina. Journal of Paleontology.

[35] Keupp HA (2012). zur Paläopathologie der Cephalopoden. Berliner paläobiologische Abhandlungen.

[36] Klug C, Fuchs D, Schweigert G, Röper M, Tischlinger H (2015). New anatomical information on arms and fins from exceptionally preserved *Plesioteuthis* (Coleoidea) from the Late Jurassic of Germany. *Swiss*. Journal of Palaeontology.

[37] Klug C, Schweigert G, Fuchs D, Kruta I, Tischlinger H (2016). Adaptations to squid-style high-speed swimming in Jurassic belemnitids. Biology Letters.

[38] Klug C (2019). Anatomy and evolution of the first Coleoidea in the Carboniferous. *Nature Communications*. Biology.

[39] Fuchs D, Klinghammer A, Keupp H (2007). Taxonomy, morphology and phylogeny of plesioteuthidid coleoids from the Upper Jurassic (Tithonian) Plattenkalks of Solnhofen. Neues Jahrbuch für Geologie und Paläontologie, Abhhandlungen.

[40] Boucot, A. J. Evolutionary paleobiology of behavior and coevolution. 690 pp. Elsevier Amsterdam Oxford-NewYork-Tokyo, (1990).

[41] Jenny D (2019). Predatory behavior and taphonomy of a Jurassic belemnoid coleoid (Diplobelida, Cephalopoda). Scientific Reports.

[42] Wild, M. *Tanystropheus longbardicus* (Bassani) (Neue Ergebnisse). - In: Kuhn-Schnyder, E., &Peyer, B.: Die Triasfauna der Tessiner Kalkalpen XXIII. *Schweizerische Paläontologische Abhandlungen* **95**, 1–162 (1973).

[43] Pinna G, Arduini P, Pesarini C, Teruzzi G (1985). Some controversial aspects of the morphology and anatomy of *Ostenocaris cypriformis* (Crustacea, Thylacocephala). Transactions of the Royal Society of Edinburgh.

[44] Brinkmann W (2004). Mixosaurier (Reptilia, Ichthyosaurier) mit Quetschzähnen aus der Grenzbitumenzone (Mitteltrias) des Monte San Giorgio (Schweiz, Kanton Tessin). Schweizerische Paläontologische Abhandlungen.

[45] Klinghardt F (1932). Über den methodischen Nachweis der Eingeweide bei fossilen Tintenfischen. Paläontologische Zeitschrift.

[46] Bandel K, Leich H (1986). Jurassic Vampyromorpha (dibranchiate cephalopods). Neues Jahrbuch für Geologie und Paläontologie, Monatshefte.

[47] Haas W (2002). The Evolutionary History of the Eight-armed Coleoidea. Abhandlungen der Geologischen Bundesanstalt (Vienna).

[48] Schweigert G, Dietl G (2010). The Coleoidea of the Upper Kimmeridgian Nusplingen Lithographic Limestone (Upper Jurassic, SW Germany) – diversity, preservation and palaeoecology. Ferrantia.

[49] Massare JA (1987). Tooth morphology and prey preference of Mesozoic marine reptiles. Journal of Vertebrate Paleontology.

[50] Carroll, R. L. & Wild, R. Marine members of the Sphenodontia. In: *In the shadow of the dinosaurs: early Mesozoic tetrapods* (eds. Fraser, N. C. & Sues, H.-D). 70–83. Cambridge, Cambridge University Press (1994).

[51] Keupp, H. *Ammoniten – paläobiologische Erfolgsspiralen*. Thorbecke Verlag, Stuttgart (2000).

[52] Kemp R (2001). Generation of the Solnhofen tetrapod accumulation. Archaeopteryx.

[53] Keller T (1977). Fraβreste im süddeutschen Posidonienschiefer. Jahreshefte der Gesellschaft für Naturkunde in Württemberg.

[54] Barthel, K. W., Swinburne, N. H. M. & Morris, S. C. Solnhofen: A study in Mesozoic palaeontology. 236 pp. Cambridge University Press, Cambridge (1990).

[55] Kriwet, J. & Klug, S. A new Jurassic cow shark (Chondrichthyes, Hexanchiformes) with comments on Jurassic hexanchiform systematics. *Swiss Journal of Geosciences* **104**, S107–S114 (2011).

[56] Rauhut OWM, Heyung AM, López-Arbarello A, Hecker A (2012). A new rhynchocephalian from the Late Jurassic of Germany with a dentition that is unique amongst tetrapods. PLoS One.

[57] Foffa D, Young MT, Stubbs TL, Dexter KG, Brusatte SL (2018). The long-term ecology and evolution of marine reptiles in a Jurassic seaway. Nature Ecology & Evolution.

[58] McCurry MR (2019). The repeated evolution of dental apicobasal ridges in aquatic-feeding mammals and reptiles. Biological Journal of the Linnean Society.

[59] Foffa D, Johnson MM, Young MT, Steel L, Brusatte SL (2019). Revision of the Late Jurassic deep-water teleosaurid crocodylomorph *Teleosaurus megarhinus* Hulke, 1871 and evidence of pelagic adaptations in Teleosauroidea. PeerJ.

[60] Wellnhofer P (1975). Die Rhamphorhynchoidea (Pterosauria) der Oberjura-Plattenkalke Süddeutschlands, Teil III: Palökologie und Stammesgeschichte. Palaeontographica Abteilung A.

[61] Bonde N, Leal MEC (2015). The detailed anatomy of *Rhamphorhynchus* II: braincase pneumatics and jaws.. Historical Biology.

[62] Fastnacht, M. Jaw mechanics of the pterosaur skull construction and the evolution of toothlessness. PhD thesis: Johannes-Gutenberg-Universität Mainz, Mainz (2005).

[63] Wellnhofer, P. *The Illustrated Encyclopaedia of Pterosaurs*. 192 pp. Salamander Books, London (1991).

[64] Veldmeijer, A. J., Signore, M. & Bucci, E. Predator-prey interaction of Brazilian Cretaceous toothed pterosaurs: a case example. In: *Predation in Organisms- A Distinct Phenomenon* (ed. Elena, A.M.T.). Springer-Verlag, Berlin, 295–308 (2007).

[65] Witton, M. P. Pterosaurs in Mesozoic food webs: a review of fossil evidence. In: *New Perspectives on Pterosaur Palaeobiology* (eds. Hone, D. W. E., Witton, M. P. & Martill, D. M.). **455**, 7–23. Geological Society Special Publications, London (2018).

[66] Clements T, Colleary C, De Baets K, Vinther J (2016). Buoyancy mechanisms limit preservation of coleoid cephalopod soft tissues in Mesozoic Lagerstätten. Palaeontology.

[67] Janicke V (1970). Ein *Strobilodus* als Speiballen im Solnhofer Plattenkalk (Tiefes Untertithon, Bayern). Neues Jahrbuch für Geologie und Paläontologie..

[68] Bakker, R. T. *The Dinosaur Heresies*. 481 pp. William Morrow and Co, New York (1986).

[69] Humphries S, Bonser RH, Witton MP, Martill DM (2007). Did pterosaurs feed by skimming? Physical modelling and anatomical evaluation of an unusual feeding method. PLoS Biology.

[70] Witton MP, Naish D (2008). A reappraisal of azhdarchid pterosaur functional morphology and paleoecology. PLoS One.

[71] Witton MP, Naish D (2015). Azhdarchid pterosaurs: water-trawling pelican mimics or “terrestrial stalkers”?. Acta Palaeontologica Polonica.

[72] Hone DWE, Henderson DM (2014). The posture of floating pterosaurs: ecological implications for inhabiting marine and freshwater habitats. Palaeogeography, Palaeoclimatology, Palaeoecology.

[73] Bennett SC (1995). A statistical study of *Rhamphorhynchus* from the Solnhofen limestone of Germany: year-classes of a single large species. Journal of Palaeontology.

